# Artery first approach in robotic pancreatoduodenectomy: right-sided uncinate first technique

**DOI:** 10.1007/s13304-025-02384-x

**Published:** 2025-09-30

**Authors:** Philipp Seeger, Asmus Heumann, Thilo Hackert, Faik Güntac Uzunoglu

**Affiliations:** Department of General, Visceral and Thoracic Surgery, University Medical Center Hamburg-Eppendorf, Martinistraße 52, 20251 Hamburg, Germany

**Keywords:** Robotic surgery, Pancreatic surgery, Triangle, Uncinate first, Artery first, Pancreatic cancer

## Abstract

Several advances have been made since the first days of pancreatic surgery and the Whipple–Kausch procedure: the artery-first approach and TRIANGLE resection for pancreatic cancer of the head have been reported in different variations for open surgery to achieve a complete R0 resection. Since this is one of the main factors for oncologic outcome, it is essential to transfer these techniques to the newest iteration of surgical evolution: robotic pancreatoduodenectomy has shown its comparability to the open approach in the last few years. In this paper, we describe our technique of robotic uncinate-first approach for superior mesenteric artery dissection and provide short-term outcomes from a small patient series. The robotic right-sided uncinate-first approach is feasible and allows safe transfer of oncologic vascular dissection techniques to the minimally invasive setting.

**Supplementary Information:**

The online version contains supplementary material available at 10.1007/s13304-025-02384-x.

## Introduction

Partial pancreatoduodenectomy (PD) is the only curative therapy for ductal adenocarcinoma (PDAC) of the pancreatic head [1]. The surgical treatment of other entities, such as rectal carcinoma, has benefitted in the past years from standardized peritumoral resection, namely complete mesorectal excision (CME), which significantly improved oncologic outcomes [2]. The exact mode of local infiltration is known for PDAC, especially along perineural tissue spreading from the mesopancreas region to the celiac trunk and the superior mesenteric artery (SMA) [3]. Since infiltration of peritumoral soft tissue (both lymphatic and perineural) has been identified as an essential factor of oncologic outcome and recurrence [4, 5], control of the SMA is one of the critical aspects of modern pancreatic surgery. This is why one of the most common techniques for resection in open surgery is the *artery-first approach*, first described by Pessaux et al. in 2006 [6]. Apart from a complete resection of lymphatic tissue, moving the dissection along the intrapancreatic SMA to the first phase of resection brings several other advantages: An aberrant right hepatic artery (RHA) originating from the SMA in 15–20% of patients [6] can be well preserved if approached from its origin. By early ligation of the inferior pancreaticoduodenal vessels, blood loss can be reduced. An infiltration of the SMA not visible in preoperative CT scans can be recognized early so that further resection can be stopped in favor of neoadjuvant treatment. Otherwise, patients suffer from perioperative morbidity with impaired survival because of positive resection margins [6]. Further adding to the complete mesopancreatic resection of PDAC, the TRIANGLE operation has been described, which includes soft tissue resection in the triangle between the celiac trunk, portal vein, and SMA [7, 8].

Another development in PD is the advent of robotic systems during the last 15 years, which has the potential to provide a minimally invasive approach with similar oncologic outcomes for a procedure whose main downside is its relatively high morbidity. Compared with the laparoscopic technique, advantages include a shorter learning curve [9] and better local tissue and bleeding control. An additional important factor is the enhanced ergonomics. Although more extensive randomized controlled trials could not show significant benefits in blood loss, length of stay, or oncologic outcomes, an advantage for fit patients with standard anatomy and locally limited disease can be expected [10].

Different techniques have been reported for minimally invasive (including robotic) artery-first dissection in PD; preparation is possible from the anterior, posterior, and left side, while the most widely used is the right-sided approach [11, 12]. This technique stems from the uncinate-first approach introduced by Hackert et al. [13 in 2010].

Whether the principles and benefits of this approach can be transferred to the robotic setting remains a relevant and yet incompletely answered question. In this article, we present our translation of a right-sided, uncinate-first approach combined with TRIANGLE resection to the robotic platform for pancreaticoduodenectomy and discuss the clinical outcomes achieved at our center in a small case series.

## Methods

### Equipment

**Fig. 1:**
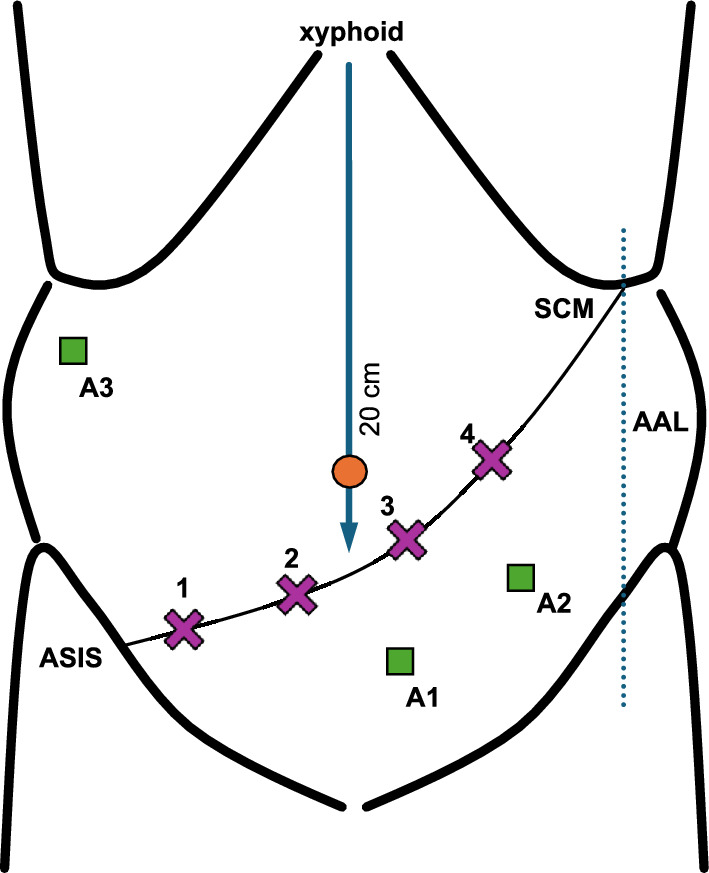
Trocar positions. 1–4: robotic trocars, placed on a line between the intersection of the left subcostal margin (SCM), the anterior axillary line (AAL), and the anterior superior iliac spine (ASIS). The Line is angled to intersect the median Line about 20 cm from the xyphoid. 1—bipolar forceps, 2—camera, 3—vessel sealer/needle holder/monopolar scissors, 4—tip-up, A1/2—assistant trocars, A3—liver retractor. (adapted according to Hackert and Croner [23])

We use the da Vinci^®^ Xi system (dVss) (Intuitive Surgical, Sunnyvale, California, USA). Instruments commonly used at our institution are bipolar forceps, vessel sealer^®^ (all at robotic trocar no. 3), and tip-up (no. 4) (Fig. 1). Traction is primarily achieved using the fourth robotic arm equipped with the tip-up fenestrated grasper, while dissection and energy application are performed through the first and third arms. Pneumoperitoneum is typically maintained at 14 mmHg. The table assistant plays an active role during key steps of the procedure, including suction and irrigation, providing additional traction with a fenestrated grasper, and applying clips when necessary.

### Patient selection

Criteria for patients suitable for robotic pancreaticoduodenectomy (RPD) are [12]:

- no prior major abdominal surgery
- ASA < 3
- BMI < 35 kg/m^2^

Patients with venous tumor contact of less than 180° or prior neoadjuvant chemotherapy are not categorically excluded from robotic evaluation and are routinely considered for the minimally invasive approach. In contrast, cases with clear preoperative evidence of vascular infiltration exceeding 180° or arterial involvement are currently reserved for open surgery due to the anticipated need for complex vascular reconstruction.

### Patient position and trocar placement

The patient is positioned with the legs spread apart in a 15°–20° anti-Trendelenburg position. The patient’s longitudinal axis is rotated by 10°–15° so that the right half of the body is elevated. The four robotic trocars are placed on an angled line below the navel, ascending from the right caudally (anterior superior iliac spine) to the left cranially (left subcostal margin); two assistant trocars are added caudally to this line, and a third one for a liver retractor in the upper right abdomen (Fig. 1).

### Exposure

**Fig. 2:**
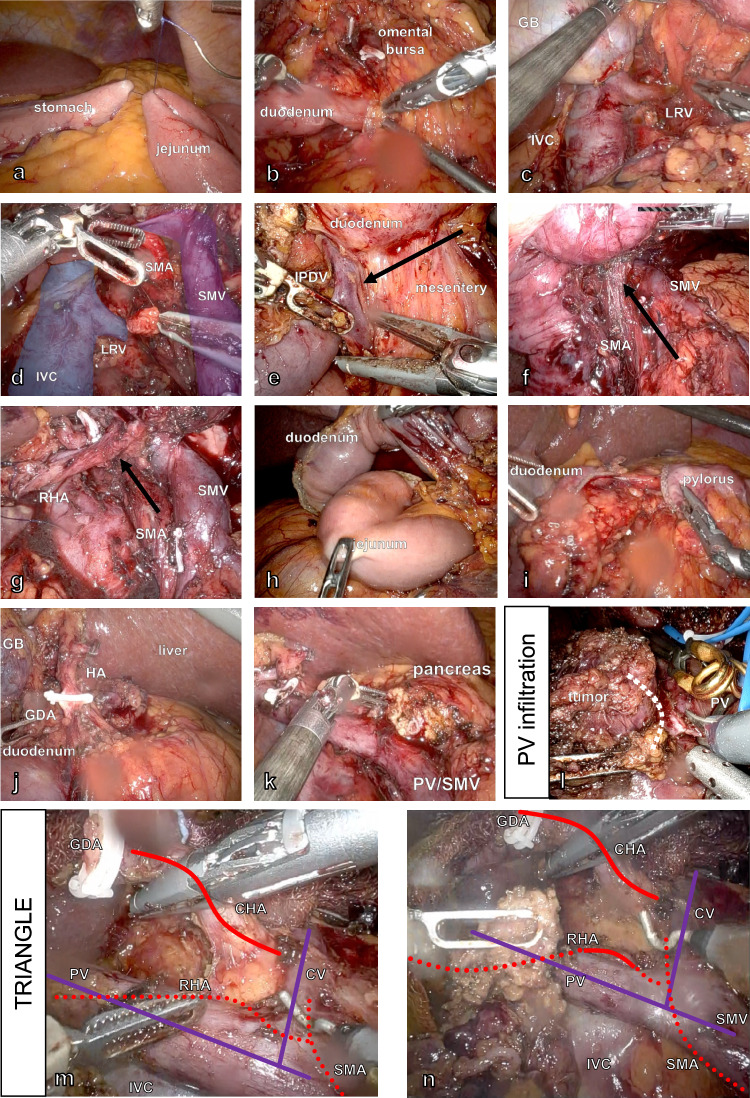
Important steps of robotic uncinate first approach and triangle procedure. **a** Fixing a jejunal loop to the stomach about 60 cm from Treitz. **b** Accessing the omental bursa via the gastrocolic ligament, the right gastroepiploic vessels have been ligated using clips. **c**, **d** Extended Kocher mobilization until the superior mesenteric artery (SMA) and the left renal vein (LRV) are visible (SMV—superior mesenteric vein, VCI—inferior vena cava). **e** The mesentery is dissected towards its origin; the inferior pancreatoduodenal vein is ligated (IPDV). **f** Dissection along the SMA continues toward its proximal origin. **g** An aberrant right hepatic artery (RHA) is preserved, and adherent tissue is dissected. **h** After moving the jejunum to the right around the mesentery, it is transected using a linear stapler. **i** Transection of the duodenum distally from the pylorus. **j** View of the hepatoduodenal ligament after lymphadenectomy. The gastroduodenal artery (GDA) has been clipped but not cut (HA—hepatic artery, GB—gall bladder). **k** The gland is cut on the portomesenteric axis (PV—portal vein). **l** In the case of minor PV tumor infiltration, a wedge (white dotted line) is resected from the PV. **m**, **n** Soft tissue in the triangle between the common hepatic artery (CHA)/celiac trunk, PV, and SMA is resected for complete mesopancreas resection (CV—coronary vein)

After ruling out peritoneal metastasis, the jejunal loop of the gastroenterostomy (100 cm from Treitz) can be fixed to the large curvature of the stomach with a suture to facilitate the subsequent placement of the gastrojejunostomy (Fig. 2a). Access to the omental bursa is then achieved through the gastrocolic ligament, which is dissected to the right until the right colic flexure is mobilized. The right gastroepiploic vessels are ligated (Fig. 2b). Moving to the right, the duodenum and the pancreatic head are mobilized from the retroperitoneum until the left renal vein (LRV) and the SMA are visualized (Fig. 2c, d) to rule out extended tumor involvement. The dorsal mesopancreatic tissue containing the lymph nodes of group 17 is kept adherent to the pancreatic head specimen.

### Uncinate first approach

With the duodenum and the pancreatic gland pulled to the right, we are performing an uncinate first approach to clear the lymphatic and nerval tissue between the SMA, SMV, and uncinate process of the pancreas. The first jejunal vein (FJV) and its origin from the SMV are located by dissecting the jejunal mesentery. Dissection starts here towards the distal jejunal mesentery; the inferior pancreaticoduodenal vein (IPDV) is ligated using surgical clips or an energy device (Fig. 2e). Now the SMA becomes visible by pulling the SMV to the left (Fig. 2f). The lymphatic tissue (lymph nodes 14b–d) and the nerve plexus adjacent to the adventitia of the SMA are dissected carefully towards the proximal origin of the SMA. The inferior pancreatoduodenal artery (IPDA) is ligated using surgical clips. If present, an aberrant right hepatic artery from the SMA must be preserved during these steps (Fig. 2g).

### Resection

After securing resectability along the SMA, the ligament of Treitz and the jejunal mesentery are divided, and the jejunal loop can now be pulled to the right of the mesenteric root. It is then transected using a linear stapler (Fig. 2h). The duodenum’s first part is transected as well distally from the pylorus using a linear stapler (leaving the 6th infrapyloric lymph node group on the specimen) (Fig. 2i). Preparation commences at the upper pancreatic border with lymphadenectomy along the hepatic artery (HA) to the hepatoduodenal ligament (L5, 8, 12b/c). The gastroduodenal artery (GDA) is ligated using surgical clips (Hem-o-lok^®^, Teleflex Medical, Research Triangle Park, North Carolina, USA) (Fig. 2j) after checking perfusion on the hepatic artery during test-clamping the GDA. The same ligation method is used on the common bile duct, and the distal duct margin is checked for malignant cells in frozen section histology. A cholecystectomy can be performed before or after these steps, as it sometimes can help to put tension on the hepatoduodenal ligament structures. The gland should now be thoroughly dissected from the portomesenteric axis. It can be cut using monopolar scissors without coagulating the pancreatic duct (Fig. 2k).

### Tumor involvement of portal vein or SMV

If the tumor includes part of the portal vein (PV) or SMV, a partial or complete venous resection can be carried out during the above steps after the complete dissection of the specimen from the SMA. Secure clamping of the proximal and distal parts of the infiltrated vein is essential to avoid blood loss and loss of overview. If necessary, clamping of the SMA is critical to prevent venous congestion of the bowel during prolonged reconstruction time [12]. We frequently use bovine pericardium for patch reconstruction after wedge resection (Fig. 2l) or Gore-Tex interposition grafts if direct end-to-end anastomosis is not possible.

### TRIANGLE resection

After the dissection, the specimen is put in a retrieval bag. To ensure complete resection of the mesopancreatic tissue, including lymph node groups 9 and 14a, extended resection between the celiac trunk, portal vein, and AMS (Fig. 2m, n) is carried out according to the TRIANGLE procedure [7].

### Reconstruction

After a complete resection and a report of negative margins in the frozen section, reconstruction is performed according to individual standards. We use a modified Blumgart pancreatojejunostomy [14], continuous hepaticojejunostomy, and stapled gastrojejunostomy with a second suture row.

### Patient cohort and data collection

We retrospectively analyzed a consecutive cohort of patients undergoing RPD with an artery-first approach along the SMA for histologically confirmed PDAC between 06/2021 and 06/2025 at University Medical Center Hamburg-Eppendorf. All procedures were performed by a dedicated robotic HPB surgical team following a standardized technique.

Patient demographics, perioperative variables, tumor characteristics (including tumor size, T-stage, nodal involvement, and vascular contact), and postoperative outcomes were extracted from electronic medical records. Complications were graded according to the Clavien–Dindo classification. Resection margin status was defined as follows: R0 wide (CRM−) if the tumor was more than 1 mm from the resection margin, R0 narrow (CRM+) if the tumor was within 1 mm but not at the margin, and R1 if tumor cells were present directly at the margin.

Descriptive statistics were reported as medians with ranges or counts. All data were anonymized prior to analysis. This study was approved by the institutional ethics committee (IRB No. PV3548).

## Results

Between 2021 and 2025, 10 patients underwent robotic pancreatoduodenectomy for PDAC using a superior mesenteric artery (SMA)-first, right-sided uncinate-first approach (Table 1). Median patient age was 70 years (range 57–76), with a female-to-male ratio of 6:4 and a median BMI of 22 (range 20–31). Most patients were ASA III. Prior abdominal surgery was present in four cases, including appendectomy (*n* = 1), cholecystectomy (*n* = 2), and gynecologic procedures (*n* = 2). Preoperative tumor staging revealed cT1 in 3 and cT2 in seven cases; two patients were cN1. Venous tumor contact (portal or superior mesenteric vein) was preoperatively suspected in seven patients. However, true vascular infiltration requiring resection was confirmed in only one case, in which a tangential portal vein resection was performed (ISGPS type 1). One patient had received neoadjuvant chemotherapy. No intraoperative conversions occurred. Median operative time was 418 min (range 354–713), with a median blood loss of 375 mL (range 200–2000). R0 resection was achieved in 8 out of 10 cases. True R1 resections occurred in two patients, involving the ventral and dorsal margins. Regarding circumferential resection margins (CRM), three patients had a positive CRM: one at the SMV groove, one at the dorsal margin, and one involving both SMV groove and dorsal margin. Median lymph node yield was 28 (range 13–36) with median 2 positive lymph nodes (0–12). Clavien–Dindo grade ≥ III complications occurred in 3 patients (30%), including two reoperations for postoperative hemorrhage. Median length of stay was 12 days (range 6–39). No 30-day mortality was observed.

**Table 1:** Case series

Robotic PD with SMA first approach		
*n* = 10, 2021–2025		
Patient characteristics (median/*n*, range)		
Age	70	(57–76)
Sex (F/M)	6/4	–
BMI	22	(20–31)
ASA	III	(II–III)
II	3	–
III	7	–
Prior abdominal surgery	4	–
Appendectomy	1	–
Cholecystectomy	2	–
Gynecologic procedures	2	–
Preoperative status (median/*n*, range)		
cT	2	(1–2)
cT1	3	–
cT2	7	–
cN1	2	–
Vascular involvement PV/SMV	7	–
Neoadjuvant chemotherapy	1	–
Operative parameters (median/*n*, range)		
Operative time (min)	418	(354–713)
Blood loss (ml)	375	(200–2000)
Conversions	0	–
Vascular resection	1	PV wedge
Pathological outcomes (median/*n*, range)		
R0	7	–
CRM+	3	2× SMV, 2× dorsal
R1	2	–
Dorsal	1	–
Ventral	1	–
pT	2	(1–3)
pT1	1	–
pT2	7	–
pT3	2	–
pN	1	(0–2)
pN0	2	–
pN1	5	–
pN2	3	–
Lymph node yield	28	(13–36)
Positive lymph nodes	2	(0–12)
Postoperative outcomes (median/*n*, range)		
Clavien–Dindo ≥ 2	6	(2–3b)
2	4	Chyle leak
3b	2	Hemorrhage TC/GDA
POPF ≥B	0	–
DGE	0	–
LOS	11	(6–39)
30-day mortality	0	–

Summarized data regarding patient characteristics, preoperative status, intraoperative parameters, postoperative and pathological outcomes of 10 patients from 2021 to 2025, single center

*PD* pancreatoduodenectomy, *SMA* superior mesenteric artery, *PV* portal vein, *SMV* superior mesenteric vein, *TC* coeliac trunc, *GDA* gastroduodenal artery, *POPF* postoperative pancreatic fistula, *DGE* delayed gastric emptying, *LOS* length of stay

## Discussion

Robotic surgery has become one of the standard techniques in many fields, sometimes even the “gold” standard (e.g., for rectal cancer resection [15]). Regarding pancreatic cancer, robotic surgery still needs to prove its advantages over open techniques. At the same time, potential benefits include less blood loss and a faster post-operative recovery with a shorter hospital stay [16, 17]. Since all the past advances in pancreatic surgery have been primarily made in open surgery, these techniques must be translated to the new minimally invasive approach. This paper describes our robotic uncinate first approach to the SMA, which draws its roots from conventional surgery [7, 13].

The most important advantage of the artery-first approach is achieving higher rates of R0 resection and more prolonged disease-free survival. Lin et al. [18] demonstrated this in a multicenter randomized controlled trial, showing significantly improved disease-free survival and lower locoregional recurrence in patients undergoing extended SMA dissection. While Sabater et al. [19] did not observe a clear benefit in R0 resection rates, the artery-first technique may nonetheless offer procedural advantages by providing a systematic dissection strategy, particularly in the minimally invasive setting where standardization supports reproducibility and team learning. The SMA margin is most often affected by tumor involvement in the resected specimen [20, 21]. A thorough resection of the soft tissue along the SMA maximizes the possibility of an R0 medial resection margin. In our cohort, the robotic right-sided artery-first approach resulted in negative margins at the SMA site in all cases; however, it must be noted that patients with preoperative evidence of SMA contact were excluded from robotic exploration. Our R0 resection rate of 80% is comparable to the results reported in recent randomized trials on open and robotic pancreaticoduodenectomy [10, 19], although direct comparison is limited by sample size and patient selection.

Another quality metric of oncologic resection is the extent of lymphadenectomy: Regarding RPD and OPD literature comparison, the number of harvested lymph nodes shows no significant difference in a meta-analysis of 11 non-randomized trials and the two recent randomized reports and amounts to 10–37 [10, 16, 17]. Our median lymph node yield of 28 falls within the upper range of what has been reported in high-volume centers and underscores the oncologic adequacy of the robotic TRIANGLE dissection.

A core aspect of this technique is the early identification of arterial involvement and, therefore, the chance of stopping exploration before reaching the point of no return, especially in patients without prior neoadjuvant therapy. No arterial involvement was found in our patient cohort, although venous contact was present in 7 patients and required a tangential portal vein resection in one case. Notably, no conversion to open surgery was necessary despite venous contact. In all cases with SMV contact, R0 resection at the SMV groove was achieved. CRM positivity at the SMV groove was identified in two patients. These findings suggest that venous contact does not necessarily preclude margin-negative resection. Still, margin positivity—particularly at the posterior or medial aspect—remains a well-recognized challenge in pancreatoduodenectomy, even with optimized exposure.

Operative time in our series was 418 min, which is comparable to other reports using a robotic approach. The EUROPA trial reported a mean duration of 431 min for RPD vs. 367 min for OPD [10], while Lin et al. reported shorter times in the RPD group (245 vs. 298 min) [17]. The longer duration in our cohort may reflect the learning curve and complex dissections involving venous contact but remains within the expected range for robotic pancreatic head resections.

With a major complication rate (Clavien–Dindo ≥ III) of 30%, including two reinterventions for hemorrhage and no 30-day mortality, our results are consistent with published data. Recent RCTs report severe complication rates of 22–30% for RPD, with comparable or lower mortality than OPD [10, 17]. All procedures in this series were performed by a dedicated robotic HPB team using a standardized technique. While the surgeons had substantial prior experience in open and minimally invasive pancreatic surgery, the early phase of implementing the robotic approach may have contributed to the observed morbidity. Notably, chyle leaks occurred frequently and were managed successfully with dietary restriction (MCT diet), potentially reflecting the extent of lymphadenectomy associated with the robotic dissection technique.

The technique described in this paper is one of the most widely used in minimally invasive surgery [11] since the anterior and left approaches are challenging to achieve due to limitations in camera movement. The camera is introduced from robotic port nr. 2 (Fig. 1), so the perspective on the portomesenteric axis will always be from the right. Another option to perform the soft tissue clearance is to start from the origin of the SMA towards caudally, using the posterior approach [22]. The latter approach has its advantages in case of tumor infiltration of the mesentericoportal axis, making it difficult to access the SMA during the uncinate first approach. This approach can be performed as a second step after dissecting the gastrocolic ligament and mobilizing the duodenum. If arterial involvement is detected, there has been even less “surgical damage” than in the right uncinate first approach. Therefore, the uncinate first approach and the posterior approach are the two most transferable techniques in robotic surgery. The choice of the respective techniques depends on patients’ anatomy and surgeons’ preferences. In our center, the right-sided uncinate first approach is the standard technique. During the learning curve, rather than changing between different techniques, we argue that staying with a standard technique provides more consistent results. Venous resection is also feasible in the right-sided approach as shown in our case series. While posterior and anterior artery-first strategies offer valuable alternatives, standardizing the right-sided uncinate-first approach may enhance reproducibility and facilitate team learning during the institutional implementation of robotic PD programs.

In conclusion, the open technique of uncinate-first SMA dissection and TRIANGLE clearance is safely and effectively transferable to the robotic platform, offering precise vascular control and high oncologic quality even in selected high-risk cases.

### Limitations

This study has limitations, including its retrospective design, small sample size, and absence of a control group. Longer-term oncologic outcomes such as disease-free and overall survival were not evaluated. Furthermore, results reflect a high-volume center with established robotic expertise, which may limit generalizability.

## Supplementary Information

Below is the link to the electronic supplementary material.

Supplementary file1 (DOCX 292 KB)

## Data Availability

The datasets generated and/or analyzed during the current study are not publicly available due to patient privacy and institutional regulations but are available from the corresponding author on reasonable request.
