# Supplementary material for: Artery first approach in robotic pancreatoduodenectomy: right-sided uncinate first technique
Source: Updates Surg. 2025 Sep 30;78(2):735–41. doi: 10.1007/s13304-025-02384-x (PMC13212737; doi:10.1007/s13304-025-02384-x)

**Video**

Video (4min) with audio narration and in-screen annotations showing our right-sided uncinate-first approach:

[LINK](https://drive.google.com/file/d/15ILFT95lrzL_P-EGTHL94X7_ImC4ldkD/view?usp=sharing) or https://drive.google.com/file/d/15ILFT95lrzL_P-EGTHL94X7_ImC4ldkD/view?usp=sharing


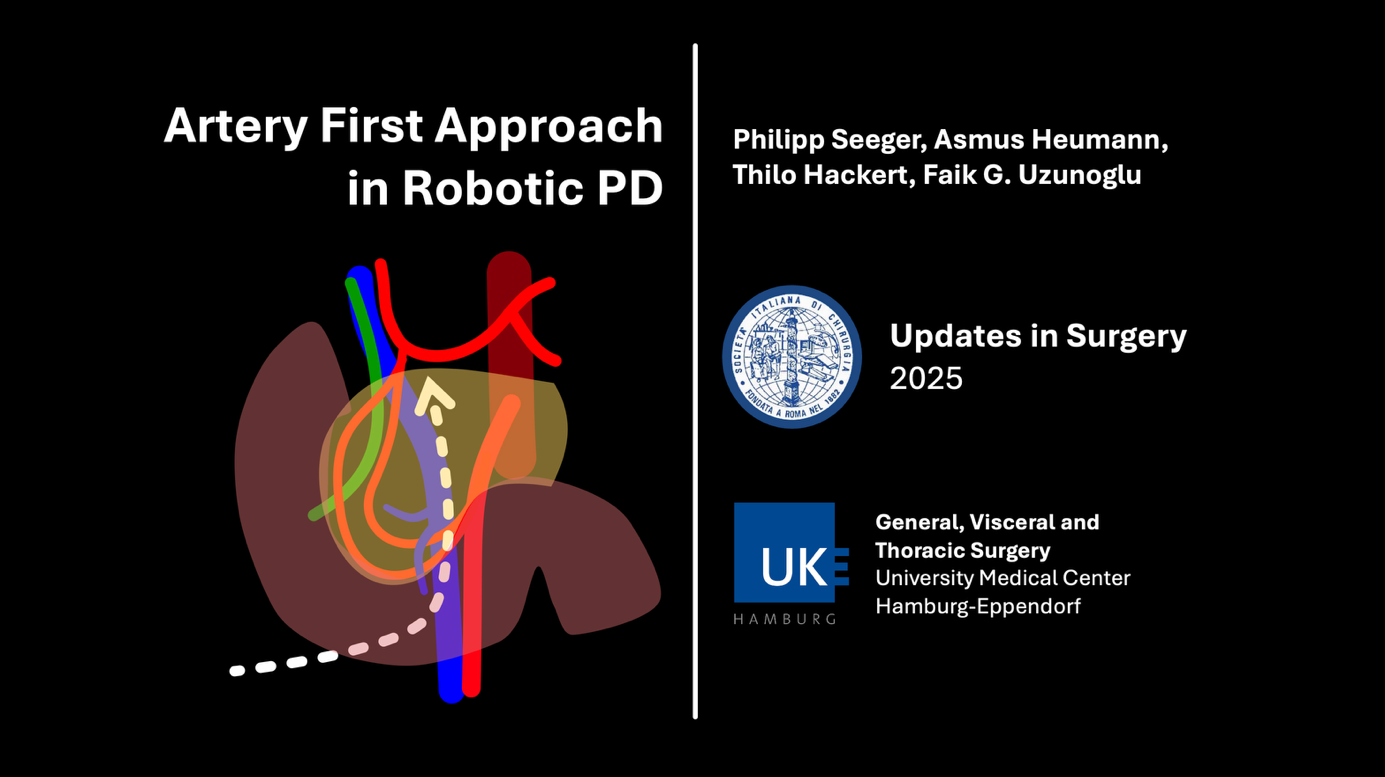

Supplement: Supplementary file 1 — Supplementary file1 (DOCX 292 KB) [file 13304_2025_2384_MOESM1_ESM.docx]
